# Pharmacological management of pediatric insomnia

**DOI:** 10.3389/frsle.2024.1389052

**Published:** 2024-06-19

**Authors:** Sakshi Dhir, Nicolette Karim, Haley Berka, Jess Shatkin

**Affiliations:** ^1^Grossman School of Medicine, New York University, New York, NY, United States; ^2^Langone Medical Center, New York University, New York, NY, United States

**Keywords:** pediatric sleep, pediatric insomnia, medications for pediatric insomnia, alternative medicine for pediatric insomnia, use of cannabis for insomnia

## Abstract

Insomnia is the most commonly reported sleep disorder among children and adolescents, impacting their cognitive, emotional, behavioral, and physical development. The prevalence of insomnia generally increases with age, often persisting into adulthood if unaddressed. Insomnia is exceedingly common among those with developmental disabilities and is frequently comorbid with a great range of psychiatric diagnoses. The COVID-19 pandemic has only increased the prevalence of insomnia among children and adolescents. Health care providers are routinely called upon to treat insomnia in the pediatric population. Psychoeducation and behavioral interventions, especially cognitive behavioral therapy for insomnia (CBT-I), remain the first line treatments, given empirical evidence for their efficacy and success in relapse prevention. However, medications are frequently employed in clinical practice, despite the fact that no medications are approved by the Food and Drug Administration (FDA) for the treatment of pediatric insomnia. This review was designed to educate and support practitioners who are treating children and adolescents who struggle with insomnia. A thorough narrative review was completed to identify all published medication studies of pediatric insomnia; the identified studies are described and then graded into four categories according to the strength of the evidence supporting their use, side effect profiles, co-morbidities, and overall risk vs. benefit of each pharmacological treatment. This review will help practitioners in making clinical decisions for their pediatric patients who suffer with insomnia.

## Introduction

Disordered sleep among children and adolescents is a public health concern, given its importance in the cognitive, emotional, and physical development and wellbeing of youth (Rolling et al., 2022). Insomnia is the most commonly reported sleep disorder within the pediatric population and is defined as difficulty falling and staying asleep, resulting in functional impairment (Badin et al., 2016). The prevalence generally increases with age (Ohayon, 2002), and if left untreated, insomnia often persists into adolescence and adulthood (Rolling et al., 2022).

Poor sleep has been repeatedly associated with an increased risk of psychiatric co-morbidities, including links between insomnia and suicide and self-harm behaviors (Winsler et al., 2015), disruption in the family environment (Cohen et al., 2018), poorer academic outcomes (Phillips et al., 2017), and threats to safety, such as motor vehicle accidents (Badin et al., 2016). Sleep difficulties have increased since COVID, and a recent meta-analysis observed a global prevalence of sleep difficulties among 46% of the pediatric population (Jahrami et al., 2022).

Psychoeducation and behavioral interventions, especially cognitive behavioral therapy for insomnia (CBT-I), remain the first line treatments, given empirical evidence for their efficacy and success in relapse prevention (Badin et al., 2016; Buckley et al., 2020). CBT-I is an evidence-based, multidimensional treatment that focuses on correcting the behavioral, environmental, psychological and physiological factors that both contribute to and perpetuate the symptoms of insomnia (Roane and Taylor, 2008). Improvements in sleep onset latency, sleep efficiency, total sleep time and wake after sleep onset have been well-established among adolescents and adults who receive CBT-I, and although some studies have found similar benefits for children, additional studies are needed (Schlarb et al., 2016; Bruni et al., 2018; Dewald-Kaufmann et al., 2019; Subotic-Kerry et al., 2023).

However, medications are frequently employed in clinical practice, despite the fact that no medications are approved by the Food and Drug Administration (FDA) for the treatment of pediatric insomnia. In a 2010 survey of 1,273 members of the American Academy of Child and Adolescent Psychiatry, 96% of physicians reported recommending at least one prescription medication, and 88% recommended at least one over-the-counter medication for insomnia in a typical month (Owens et al., 2010). Given the frequent use of medications for the treatment of insomnia in youth, this report provides graded recommendations for the pharmacological treatment of pediatric insomnia based upon a thorough review of the literature.

## Method

Authors conducted this narrative review through the utilization of PubMed using key words including “pediatric sleep, insomnia, medications for pediatric insomnia/sleep, as well as alternative medicine for pediatric insomnia/sleep, and use of cannabis for insomnia/sleep.” Peer-reviewed articles, case control studies, clinical trials, meta-analyses and randomized controlled trials for the medications described to date were reviewed by the authors. There were no specified parameters regarding the date of publication of selected articles nor any exclusion criteria in order to conduct a comprehensive and up-to-date review. Based on this information, the authors developed a medication hierarchy for the treatment for pediatric insomnia. The four categories were built upon the currently available evidence, side effect profiles, co-morbidities, and overall risk vs. benefit of each treatment option. This hierarchy is designed to serve as a reference for providers when making clinical decisions for the treatment of pediatric insomnia. The categories were formed in order to summarize the information and to give a reference point for providers to consider for clinical decision-making in the treatment of pediatric insomnia.

## OTC medications

### Melatonin and melatonin agonists

Melatonin is a naturally occurring hormone produced from the amino acid tryptophan and secreted in darkness by the pineal gland that is largely responsible for regulation of circadian rhythm and sleep-wake cycles (Rolling et al., 2022). Melatonin is available for purchase in many countries in both immediate and extended-release forms and is currently the most frequently prescribed hypnotic for pediatric patients (Boafo et al., 2020; Delrosso et al., 2021; Wesselhoeft et al., 2021), with data from the Norwegian prescription database indicating a 3–5 fold increase in the use of off-label melatonin in the pediatric population between 2004 and 2011 (Hartz et al., 2012). Melatonin is the most thoroughly studied treatment of insomnia for youth with neurodevelopmental disorders, based, in part at least, upon the hypothesis that abnormalities in melatonin secretion may be responsible for the high rates of insomnia and circadian rhythm abnormalities among those with autism spectrum disorders (ASD; Buckley et al., 2020). Numerous randomized clinical trials have demonstrated the efficacy of melatonin among children and adolescents with autism spectrum disorder in increasing total sleep time and decreasing sleep latency (Gringras et al., 2017; Hayashi et al., 2022; Xiong et al., 2023). Melatonin appears to be generally safe with long-term use of up to 2 years (Maras et al., 2018; Malow et al., 2021).

While no specific formulations have received FDA approval for pediatric insomnia in the United States, one extended-release formulation (Slenyto) has been approved in the European Union for the treatment of insomnia in children (ages 2–18) with ASD and Smith Magenis syndrome. Tablets are available by prescription in 1 and 5 mg preparations, and the recommended dosing range is 2–10 mg taken 30 min before bedtime (Institute for Quality and Efficiency in Health Care, 2019).

Several randomized controlled trials suggest that melatonin is also safe and often effective for short-term use in typically developing children and adolescents (Smits et al., 2003; Jalilolghadr et al., 2022). Further, a consensus panel of pediatric sleep clinicians in the US has advised that 1–5 mg of melatonin taken 30–60 min before bedtime may be beneficial among youth who are refractory to behavioral interventions (Goldman et al., 2021). The American Academy of Sleep Medicine, however, has advised that there is weak evidence in favor of using strategically timed melatonin for the treatment of Delayed Sleep-Wake Phase Disorder in children and adolescents without psychiatric comorbidites to advance sleep onset or decrease sleep latency (Auger et al., 2015). Additionally, a 2023 meta-analysis published by the Danish Health Authority of eight randomized, controlled trials in typically developing youth with idiopathic insomnia found only a modest decrease in sleep latency and a similarly modest increase in total sleep time, but no significant effect on sleep quality or daytime functioning was observed. An increased prevalence [RR (relative risk) 3.44, 95% CI 1.25–9.24] of non-serious adverse events, such as headache, nausea, and mood changes among those taking melatonin (vs. placebo) was observed in this study, but the dropout rate was not significantly elevated (Edemann-Callesen et al., 2023). Recommendations for cautious use and short duration of treatment are given. Finally, although the International Pediatric Sleep Association's (IPSA) Melatonin Task Force has advised that developmentally disabled children 2 years-of-age and older can generally be prescribed 2–10 mg of melatonin safely and with some expectation of benefit (International Pediatric Sleep Association Melatonin Task Force, 2023) the IPSA has not issued a statement on the use of melatonin for adolescents with delayed sleep phase disorder or in typically developing children with insomnia.

For patients who have not benefitted from behavioral strategies/CBT-I, we recommend melatonin as a first line medication for pediatric insomnia, given the relatively robust evidence for its safety and efficacy. Based on what is known about melatonin's efficacy in children with neurodevelopmental disorders, it deserves special consideration among this population, although it is a reasonable option in typically developing children as well. Physicians may refer to dosing guidelines in Table 1 for further information on using melatonin in practice. We recommend that patients prescribed melatonin for insomnia should continue to observe good sleep hygiene and utilize behavioral strategies while using the medication for optimal results. Prescribers are advised that because melatonin and other dietary supplements are not regulated by the FDA, the precise amount of melatonin within the tablets is not guaranteed. Numerous studies have found greatly varying amounts of active ingredient or its entire absence, along with the presence of other, unspecified ingredients such as serotonin, within tablets housed within the same bottle of melatonin (Erland and Saxena, 2017; Cohen et al., 2023). Because dietary supplements are regulated under the Dietary Supplement Health and Education Act and not by the FDA, consumers are advised to purchase only those products marketed as pharmaceutical grade.

**Table 1:** Comparison of pharmacological interventions for pediatric insomnia.

**Medication**		Dose (mg)^*^	Approximate time to peak (hours)^*^	Approximate half-life (hours)^*^	**Co-morbid conditions to consider as a guide for medication choice**	**Side effect profile**
Melatonin	Instant Release (IR)	1–10 g	IR: 2–240 min	IR: 20–30 min	ASD/NDD, post-concussive syndrome	Headache, nausea, and cognitive/mood changes (typically mild)
	Prolonged Release (PR)		PR: 2 h	PR: 3.5–4 h		
Ramelteon		3–8 mg	0.5–1.5 h	1–2.6 h	-	Dizziness, somnolence, and nausea (mild)
Alpha 2 agonists	Clonidine IR	5–10 mcg/kg or 25–50 mcg initial, titrating in 25 mcg increments to max based on weight (0.2–0.4 mg)	1–3 h	~6 h	ADHD	Bradycardia, hypotension, sedation, dizziness, and rebound hypertension on discontinuation
	Clonidine ER		7–8 h			
	Guanfacine IR	1–4 mg (ADHD)	2.6 h	~17 h		
	Guanfacine ER		5 h	14–18 h		
Antihistamines	Diphenhydramine	0.5–1 mg/kg or 12.5–50 mg	2 h	5 h	Atopic dermatitis, sleep bruxim	Drowsiness, dry mouth, urinary retention, blurred vision, QT prolongation (hydroxyzine), and paradoxical agitation
	Hydroxyzine	12.5–25 mg		5–9 h		
Antidepressants	Trazodone	Initial 0.75–1 mg/kg/day or 25–50 mg at bedtime	0.5–1.5 h; up to 2.5 h with food (adult data)	5–9 h (adult data)	Mood disorders, anxiety disorders	Dry mouth, nausea, vomiting, dizziness, drowsiness, fatigue, headache, and nervousness
	Doxepin	3–6 mg	3.5 h	15 h		
	Mirtazapine	N/A	2 h (adult data)	20–40 h (adult data)	ASD	
Gabapentin		5–15 mg/kg	-	-	-	Agitated awakenings when increased to 15 mg/kg. Feeling “wired” and having difficulty falling asleep on initiation of gabapentin
Benzodiazepines	Clonazepam		1–4 h	22–33 h	Sleep terrors, sleep walking	Impaired explicit memory and short term anterograde amnesia, drowsiness, fatigue, ataxia, psychomotor impairment, withdrawal syndrome particularly following abrupt or overly rapid discontinuation following regular use.
Z-drugs	Zolpidem	0.25 mg/kg/dose	1.1 h	2.3 h	ADHD (limited data available)	Dizziness, drowsiness, headache, nausea, anxiety, and hallucinations
Suvorexant		10–20 mg	0.5–6 h	15 h	-	Abnormal dreams, dizziness, drowsiness, headache, dry mouth, and diarrhea
Quetiapine		25–50 mg	-	-	Bipolar disorder	At higher doses, metabolic effects, anticholinergic effects, cardiac effects, extrapyramidal symptoms. Limited findings at low dose.
					ICU associated- Delirium	
					Schizophrenia	
Iron (Ferrous sulfate)		3–6 mg/kg/day	5–10 days (adult date)	-	ASD	Nausea, darkening of stool, abdominal pain, constipation, vomiting, and diarrhea
		1–2 mg/kg/day up to 6 mg/kg/day			Iron deficient	
L-theanine		400 mg	0.5–2 h	54–78 min	ADHD	N/A

^*^Dose, approximate time to peak (hours), and approximate half-life (hours) obtained from UpToDate.com (accessed January 20, 2024).

### Antihistamines

Antihistamines (H1 inverse agonists) induce sleep by suppressing the activity of histamine, a wake-promoting neurochemical, in the brain. Diphenhydramine has been included in the US Food and Drug Administration (FDA's) Final Drug Monograph for OTC use, and doxylamine (Unisom) is FDA approved for the treatment of insomnia in adults (Culpepper and Wingertzahn, 2015). Sedating antihistamines are routinely prescribed off-label to youth with insomnia (Bruni et al., 2018; Wesselhoeft et al., 2021), but the evidence in support of antihistamines for pediatric insomnia is severely limited. One double-blind, placebo-controlled crossover clinical trial of diphenhydramine at doses of 1 mg/kg in 50 children with sleep disorders found significantly reduced sleep latency and number of awakenings in those taking diphenhydramine as compared to placebo by parent report (Russo et al., 1976). Conversely, a randomized, double-blind clinical trial assessing the efficacy of diphenhydramine at 1 mg/kg in reducing nighttime awakenings in infants aged 6–15 months was terminated after showing no benefit over placebo (Merenstein et al., 2006).

Antihistamines such as diphenhydramine (Benadryl) have the benefit of being easily accessible, and their over-the-counter status may give parents the impression that they are benign; however, based on the paucity of information available in the pediatric population, we recommend that any use of this medication as a sleep aid be conservative and time-limited. Parents should be made aware of potential side effects including next-day sedation, anticholinergic effects, and risk of paradoxical agitation. This, and further dosing guidance, can be found in Table 1.

## Prescription medications

### Alpha agonists

Alpha agonists are often prescribed by US physicians for the treatment of pediatric insomnia (Schnoes et al., 2006), although there is limited evidence for this indication. The strongest support lies with clonidine, but even here the research is limited to retrospective chart reviews and case studies and only among patients with ADHD (Wilens et al., 1994; Prince et al., 1996; Ming et al., 2008). Dosages of 0.05–0.1 mg at bedtime have been reported to reduce sleep latency and improve sleep efficiency (Ming et al., 2008). Fewer studies have assessed guanfacine for the treatment of pediatric insomnia, but among this research, there is no evidence of benefit. One randomized, double-blind, placebo-controlled trial of extended release guanfacine administered in the morning in children with ADHD was terminated early after REM and total sleep time were found to be significantly reduced in the treatment group compared to placebo (Rugino, 2018). Similarly, a randomized, placebo-controlled trial of extended release guanfacine (1–4 mg) in children with comorbid ASD and ADHD that assessed change in sleep as a secondary outcome found no statistical separation between treatment and placebo groups (Politte et al., 2018).

In patients with ADHD who are struggling with insomnia (especially in the context of worsening or “rebound” symptoms in the evenings), clonidine may be preferentially used over guanfacine to maximize benefit for sleep. However, there is insufficient evidence to recommend its use in the healthy pediatric population, especially in light of potential side effects including hypotension, dizziness, and risk of rebound hypertension.

### Antidepressants

Antidepressants are occasionally prescribed for the treatment of pediatric insomnia, given that some have sedative side effects. While the tricyclic antidepressant doxepin has been FDA approved in low doses for the treatment of insomnia in adults, there is no FDA approved antidepressant for pediatric insomnia (Shah et al., 2020). Nonetheless, trazodone is frequently utilized by clinicians for pediatric insomnia when comorbid with mood and anxiety disorders. One study, however, found that youth who were prescribed trazodone in addition to a selective serotonin re-uptake inhibitor were three times more likely to express thoughts of self-harm and six times less likely to experience benefit from the antidepressant (Brent, 2008); another study also observed that combining an antidepressant with trazodone reduced the likelihood of response to the antidepressant (Sultan and Courtney, 2017), an effect hypothesized to be due to the build-up of meta-chlorophenylpiperazine (mCPP) which may lead to worsening anxiety (Shamseddeen et al., 2012). These findings have not been observed among youth taking antidepressants with other medications commonly employed for sleep, such as antihistamines. Finally, there is limited evidence that mirtazapine may be successful in treating symptoms of insomnia, anxiety, and depression in patients with comorbid ASD (Posey et al., 2001; Gupta and Gupta, 2023). Thus, given the overall limited data available for use of antidepressants for pediatric insomnia, this class of drugs should not be used as first-line treatment for pediatric insomnia (as summarized in Table 2); however, prescribers can refer to Table 1 for dosing guidance for youth with co-morbid mood disorders and insomnia.

**Table 2:** Graded recommendations for pharmacological interventions in pediatric insomnia.

**Class**	**Type of intervention**
Class I: Clear benefit demonstrated by robust evidence base	**Behavioral interventions:** such as CBT-I, which remains first line treatment given efficacy, safety and relapse prevention.
	**Melatonin:** The best studied pharmacologic intervention with the most evidence to support its use. Extensive research in the ASD/NDD population show it is well-tolerated with a minimal side effect burden and appears to be safe for long-term use. Emerging data in typically developing children with idiopathic insomnia suggests that at present it is the safest pharmacologic option in cases refractory to behavioral efforts.
Class IIa: May be beneficial in certain populations and/or for short-term use, but requires further data	**Clonidine:** May be helpful for sleep in children with ADHD, but has not been studied in a healthy, typical pediatric population; side effects (hypotension, dizziness, risk of rebound hypertension) make other options more viable in patients without ADHD.
	**Antidepressants:** Off-label use of trazodone, mirtazipine and doxepin should only be considered if there is another indication aside from insomnia guiding the medication choice. Overall, the evidence supporting the use of these antidepressants for treatment of pediatric insomnia is limited.
	**Iron:** While the current data is limited and conflicting, prescribers may consider targeting a serum ferritin level of 30–50 ng/ml or higher with iron supplementation of 1–6 mg/kg/day of elemental iron in at-risk populations (iron deficient and ASD).
	**Antihistamines:** A limited evidence base shows reduced sleep latency and improved sleep efficiency with good tolerability in short-term use, but daily use cannot be recommended given lack of long-term safety data.
Class III: Insufficient data, requiring further study	**Ramelteon:** FDA approved in adults, can be considered for use in adolescents over age 18; still requires data for younger children.
	**Gabapentin:** Limited but promising data; however, additional studies are necessary to guide treatment. Average dose of 5 to 15 mg/kg in one study of developmentally disordered children was found to be safe and effective for a majority of subjects; minimal drug interactions.
	**L-theanine:** Amino acid naturally found in green tea leaves. Limited data suggests a daily dose of 400 mg of L-theanine can potentially be safely used to improve sleep quality in children with ADHD. Additional studies are needed.
	**Orexin receptor antagonists:** Limited evidence has demonstrated that suvorexant 10–20 mg daily can improve sleep quality.
Class IV: Minimal benefit or high risk of harm	**Guanfacine:** Minimally studied, with two small trials in ADHD population showing no benefit or even worsening in reported sleep quantity/quality.
	**Benzodiazepines:** They may be of benefit to children who exhibit somnambulism, but there is insufficient data to support this claim. This minimal benefit does not outweigh the high risk of dependency and tolerance.
	**Z-hypnotics:** Minimally studied in children; while they may be good short-term treatments for insomnia in adults, the high risk of dependency and potential for paradoxical agitation in children outweigh any benefits.
	**Quetiapine:** Minimally studied in children with insomnia. Given its side effect profile and limited evidence of benefit, it should only be used for those with relevant co-morbid psychiatric conditions.
	**Marijuana: T**hough short-term use may indeed improve sleep quality and duration, and decrease sleep latency, long term use typically results in tolerance of sleep-inducing effects and increases the likelihood of sleep disturbance, poor sleep quality, and continued use of cannabis. Emerging evidence of risks of THC and the great variability in product composition make marijuana a non-viable option at this time.

### Gabapentin

While Gabapentin is not a first-line treatment for pediatric insomnia, one retrospective case series of 23 children (average age of 7.2 years, a majority with neurodevelopmental disorders) reported gabapentin to be an effective treatment for both sleep-onset and sleep maintenance insomnia in 78% of subjects by parent report (Malow et al., 2021). Parents were simultaneously educated in behavioral interventions for sleep, which may have also contributed to the benefits observed. The average starting dose was 5 mg/kg at bedtime, advanced to a maximum dose of 15 mg/kg (as summarized in Table 1), which is significantly lower than typical dosages for epilepsy. Adverse effects included agitated awakenings, which generally resolved after lowering the dosage. While this one small report was promising, further studies of gabapentin have not been performed, and thus there is insufficient evidence to recommend its use for pediatric insomnia (as summarized in Table 2).

### Ramelteon

Ramelteon is a melatonin agonist that is FDA approved for the treatment of insomnia in adults but with limited evidence in pediatric populations. A small number of case reports in children and young adults with ASD and severe developmental disability have shown potential benefit and good tolerability (Stigler et al., 2006; Miyamoto et al., 2013). As suggested in the grading recommendations in Table 2, there is currently insufficient data available to recommend its use in the general pediatric population.

## Sedatives/hypnotics

### Benzodiazepines

Benzodiazepines modulate the GABAa receptor, leading to decreased anxiety, muscle relaxation, and sedation (Griffin et al., 2013). Benzodiazepines can also decrease sleep latency and the frequency and duration of awakenings. Although statistically better than placebo in the treatment of insomnia among adults, their average effect size is small, resulting in only about 30 min of additional sleep per night (Glass et al., 2005). Unfortunately, benzodiazepines can also contribute to memory impairment, loss of coordination, and daytime sleepiness, and their chronic use is correlated with early onset dementia (He et al., 2019). Dependence and tolerance to benzodiazepines are considerable risks, and one study found that the discontinuation of benzodiazepines led to reduced sleep quality and onset, as well as increased suicide risk within 2 weeks of treatment cessation (Edinoff et al., 2021). Further, just as the dangers of benzodiazepine withdrawal in adults is well-documented, one study found that about 20% of children given benzodiazepines for sedation in an intensive care unit (typically midazolam) exhibited withdrawal effects, the symptoms of which varied and consisted of difficulty sleeping, agitation, tremors, and inconsolable crying (Edinoff et al., 2021). While benzodiazepines are FDA approved for the treatment of pediatric epilepsy, their benefit in treating pediatric insomnia has not been established. Clonazepam is the most frequently prescribed benzodiazepine for children and has been used to treat severe sleep terrors and sleepwalking (Simon and Byars, 2016). Despite the potential benefit of using benzodiazepines to treat somnambulism, there is insufficient evidence to support its use which, combined with the high likelihood of dependency and abuse potential, leads to the recommendation that benzodiazepines should not be used as a pharmacological treatment of pediatric insomnia (as summarized in Table 2).

### Non-benzodiazepines (Z-hypnotics)

Like benzodiazepines, Z-hypnotics also bind to the GABAa receptor, though more selectively and thus target the sedative effect rather than the anxiolytic effect (Drover, 2004). They have been studied in adults less extensively than benzodiazepines but have been shown to cause significant impairment, lasting up to 11 h after dosing (Brandt and Leong, 2017). Two large randomized, double-blind studies of youth with insomnia comorbid with ADHD, one of zolpidem and the other of eszopiclone, found no benefits (Blumer et al., 2009; Singh and Loona, 2013). This, combined with the high likelihood of dependence, leads to the recommendation that Z-hypnotics should not be used as a pharmacological treatment for pediatric insomnia (further summarized in Table 2).

### Orexin receptor antagonists

Orexin or hypocretin antagonists, three of which have been FDA approved for the treatment of insomnia in adults (suvorexant, lemborexant, and daridorexant), block hypothalamic orexin receptors that are important for arousal and wakefulness (Besterman and Jeste, 2023). There is limited data for their use in the pediatric population (Donskoy and Loghmanee, 2018). One small open-label study consisting of 30 patients (age range 10–20 years) reported significant improvement in the subjective sleep quality with suvorexant at a dose of 20 mg/day. Abnormal dreams were the most common side effect resulting in discontinuation (Kawabe et al., 2017). A case study of one 16-year-old male with bipolar 1 disorder given a trial of suvorexant at 10 mg nightly observed successful resolution of insomnia, along with improved sleep duration and quality (Prieto et al., 2019). Lastly, a case series of three youth and one adult with neurodevelopmental disorders reported benefit from suvorexant for both the 14-year-old adolescent and the 28-year-old young adult, who experienced significant improvements in sleep initiation and maintenance (Besterman and Jeste, 2023). The 9 and 13-year-old girls, however, experienced no benefit nor significant side effects, and their medication was discontinued. Thus, at this time, there is limited evidence that suvorexant 10-20 mg daily can improve sleep quality (as outlined in Tables 1, 2).

### Antipsychotics

Some atypical antipsychotics are being increasingly used off-label for the treatment of insomnia (Hermes et al., 2013; Thompson et al., 2016), largely due to their affinity for H1 receptors. In a cross-sectional review of 2,613 providers at a single Veteran's Administration Medical Center over a 20-month period, quetiapine was found to be the most frequently prescribed antipsychotic for sleep in adults (Hermes et al., 2013). Notably, prescriptions of quetiapine written by family physicians for sleep disturbances increased by 300% in Canada between 2005 and 2012 (Thompson et al., 2016); and a similar pattern was observed in the US where 70% of antipsychotic prescriptions written between 1996 and 2003 were for indications other than psychosis (Sankaranarayanan and Puumala, 2007). However, a 2008 US guideline for the management of chronic insomnia found insufficient evidence for atypical antipsychotics in treating insomnia, advising against their use as a first-line therapy (Thompson et al., 2016). There is even more limited data on the use of quetiapine for pediatric insomnia. Despite this fact, a 5-year observational study (2012–2016) of prescription claims for Medicaid-insured ADHD youth (ages 3–18) found that quetiapine was one of the most frequently prescribed medications for sleep (Klein et al., 2019). One case report of a 15-year-old observed that low dose quetiapine effectively treated somnambulism, possibly due to its effect in decreasing delta wave density during stage 3 sleep (Gill et al., 2011).

Some studies have shown promising results for quetiapine at 25–50 mg for the treatment of insomnia through a range of ages (Dujardin et al., 2018; Frase et al., 2018). This has also been outlined in Table 1 for dosing guidance. However, the effects of antipsychotic medications on sleep have mostly been investigated in psychiatric patients with comorbid conditions. Few small, randomized, controlled trials of adults using quetiapine at dosages of 25–100 mg at bedtime for the treatment of insomnia without co-morbid psychiatric disorders have been published, and among these studies, the data has demonstrated only non-significant trends for prolonged sleep and decreased sleep latency at best (Modesto-Lowe et al., 2021). Within the pediatric population, antipsychotic use should be restricted to only those with co-morbid psychiatric disorders (Frase et al., 2018). Quetiapine is associated with adverse metabolic events and serious adverse events include fatal hepatotoxicity, akathisia, weight gain, and restless legs syndrome (Coe and Hong, 2012; Dujardin et al., 2018). Given this side effect profile and limited evidence, its use should be limited only for those with relevant co-morbid psychiatric conditions, as summarized in Table 2.

## Dietary supplements

### Iron

Sleep disturbances in children with Attention-Deficit/Hyperactivity Disorder (ADHD) and adults with periodic limb movement disorders are correlated with low serum ferritin levels (Blackmer and Feinstein, 2016). This observation may be the result of iron's role as a co-factor in the dopamine-opiate system, which influences the sleep-wake cycle. Two parallel clinical trials conducted in a large sample of infants at high risk for iron deficiency and those with anemia observed that micronutrient iron and iron-folic supplementation increased sleep duration and reduced night waking (Kordas et al., 2009). Iron supplementation may be particularly relevant for autistic youth at high-risk for iron deficiency secondary to narrow food preferences. A retrospective, cross-sectional review of 53 pediatric ASD patients found that a median ferritin level of 24 ng/ml or lower was associated with sleep fragmentation and poor sleep efficiency (Youssef et al., 2013). Another study demonstrated improvement in restless sleep in 33 autistic youth (2–6 years of age) with oral supplementation of iron for 8 weeks (Dosman et al., 2007). In contrast, a controlled clinical trial of 20 children with ASD and low ferritin levels did not demonstrate improvement in insomnia among those treated with ferrous sulfate vs. placebo (Reynolds et al., 2020). Thus, while the available data is incomplete and conflicting, prescribers may consider treating low ferritin levels among their pediatric patients with insomnia, targeting 30–50 ng/ml with iron supplementation of 1–6 mg/kg/day of elemental iron in at-risk populations (Blackmer and Feinstein, 2016).

### L-theanine

L-theanine is an amino acid found in green tea leaves, Camellia sinensis, and is thought to alleviate stress and improve sleep through a glutamate receptor-mediated mechanism (Lyon et al., 2011; Innocenti et al., 2023). One randomized, 10-week, double-blind, placebo-controlled trial conducted with 98 boys, ages 8–12 years, with a diagnosis of ADHD observed improved sleep efficiency as measured by actigraphy but no change in sleep latency or duration at doses of 200 mg twice daily. The treatment was well-tolerated without observable side effects (Lyon et al., 2011). Thus, providers can consider a daily dose of L-theanine 400 mg to potentially improve sleep quality in youth with ADHD, although further studies are required for this to become a part of practice (as outlined in Tables 1, 2).

### Marijuana

Irregular sleep schedules have been found to correlate with frequency of substance use, and numerous studies have found that sleep problems often precede, and can be predictive of, future adolescent substance use (Babson et al., 2017). One study found that young adult cannabis users with greater past-year use had significantly worse sleep quality. Higher dosage and/or more frequent use was predictive of poorer sleep (Maple et al., 2016). Occasional endocannabinoid use generally promotes sleep by reducing sleep latency and increasing slow wave and REM sleep.

Chronic cannabis use, however, likely decreases endocannabinoid signaling and, subsequently, decreases endogenous cannabinoid effects, which results in a reduction in slow wave and REM sleep and an increase in excessive daytime sleepiness. Because slow wave and REM sleep are necessary for the efficient consolidation of long-term memory, interrupting these sleep stages can lead to significant memory difficulties (Hirvonen et al., 2011). Thus, the minimal benefit does not outweigh the high risk of harm of using marijuana and it, therefore, should not be recommended as a potential treatment for pediatric insomnia. Table 1 summarizes the aforementioned pharmacological interventions.

## Conclusion and recommendations

Limited evidence supports the use of psychopharmacological interventions for the treatment of pediatric insomnia. Psychoeducation and behavioral interventions, especially cognitive behavioral therapy for insomnia (CBT-I), remain the first line treatments, given empirical evidence for their efficacy and success in relapse prevention (Badin et al., 2016; Buckley et al., 2020). CBT-I is an evidence-based, multidimensional treatment that focuses on correcting the behavioral, environmental, psychological and physiological factors that both contribute to and perpetuate the symptoms of insomnia (Roane and Taylor, 2008). Sleep education, stimulus control, sleep restriction therapy, sleep hygiene, cognitive therapy and relaxation training are the mainstays of CBT-I treatment and can be taught to patients over a small number of sessions (Badin et al., 2016). Improvements in sleep onset latency, sleep efficiency, total sleep time and wake after sleep onset have been well-established among adolescents and adults who receive CBT-I, and although some studies have found similar benefits for children, additional studies are needed (Schlarb et al., 2016; Bruni et al., 2018; Dewald-Kaufmann et al., 2019; Subotic-Kerry et al., 2023).

A thorough risk benefit analysis should be employed when considering pharmacological interventions for pediatric insomnia. The severe detriments of sleep deprivation on the developing brain in children and the increase in risk-taking behavior in under-slept adolescents should factor into this decision, and in some cases using agents with less established evidence may be warranted (Shatkin, 2017). Practitioners are advised to involve patients and their families in the decision-making process as much as possible and to start with psychoeducation and behavioral interventions and to continue them even while using psychopharmacology as an adjunct. The limited data in this report can be used as a guide to help practitioners make rational clinical decisions.

The evidence for each of the agents has been assigned to a graded recommendation scale described in Table 2.

## Author contributions

JS: Conceptualization, Methodology, Project administration, Writing – original draft, Writing – review & editing. SD: Conceptualization, Methodology, Writing – original draft, Writing – review & editing. NK: Conceptualization, Methodology, Writing – original draft, Writing – review & editing. HB: Conceptualization, Methodology, Writing – original draft, Writing – review & editing.
